# Contaminant concentration versus flow velocity: drivers of biodegradation and microbial growth in groundwater model systems

**DOI:** 10.1007/s10532-018-9824-2

**Published:** 2018-02-28

**Authors:** Michael Grösbacher, Dominik Eckert, Olaf A. Cirpka, Christian Griebler

**Affiliations:** 1 Helmholtz Zentrum München - German Research Center for Environmental Health, Institute of Groundwater Ecology, Ingolstädter Landstrasse 1, 85764 Neuherberg, Germany; 2 Center for Applied Geoscience, University of Tübingen, Hölderlinstrasse 12, 72074 Tübingen, Germany; 3 Present Address: Ingenieurgesellschaft Prof. Kobus und Partner GmbH, Heßbrühlstrasse 21D, 70565 Stuttgart, Germany

**Keywords:** Groundwater, Biodegradation, Aromatic hydrocarbons, Natural attenuation, *Pseudomonal putida* F1, *Aromatoleum aromaticum* EbN1

## Abstract

Aromatic hydrocarbons belong to the most abundant contaminants in groundwater systems. They can serve as carbon and energy source for a multitude of indigenous microorganisms. Predictions of contaminant biodegradation and microbial growth in contaminated aquifers are often vague because the parameters of microbial activity in the mathematical models used for predictions are typically derived from batch experiments, which don’t represent conditions in the field. In order to improve our understanding of key drivers of natural attenuation and the accuracy of predictive models, we conducted comparative experiments in batch and sediment flow-through systems with varying concentrations of contaminant in the inflow and flow velocities applying the aerobic *Pseudomonas putida* strain F1 and the denitrifying *Aromatoleum aromaticum* strain EbN1. We followed toluene degradation and bacterial growth by measuring toluene and oxygen concentrations and by direct cell counts. In the sediment columns, the total amount of toluene degraded by *P. putida* F1 increased with increasing source concentration and flow velocity, while toluene removal efficiency gradually decreased. Results point at mass transfer limitation being an important process controlling toluene biodegradation that cannot be assessed with batch experiments. We also observed a decrease in the maximum specific growth rate with increasing source concentration and flow velocity. At low toluene concentrations, the efficiencies in carbon assimilation within the flow-through systems exceeded those in the batch systems. In all column experiments the number of attached cells plateaued after an initial growth phase indicating a specific “carrying capacity” depending on contaminant concentration and flow velocity. Moreover, in all cases, cells attached to the sediment dominated over those in suspension, and toluene degradation was performed practically by attached cells only. The observed effects of varying contaminant inflow concentration and flow velocity on biodegradation could be captured by a reactive-transport model. By monitoring both attached and suspended cells we could quantify the release of new-grown cells from the sediments to the mobile aqueous phase. Studying flow velocity and contaminant concentrations as key drivers of contaminant transformation in sediment flow-through microcosms improves our system understanding and eventually the prediction of microbial biodegradation at contaminated sites.

**Electronic supplementary material:**

The online version of this article (10.1007/s10532-018-9824-2) contains supplementary material, which is available to authorized users.

## Introduction

Groundwater is one of the most important resources of drinking water, accounting for 70% of public water supply in Germany. It is increasingly threatened by pollution (Bauer et al. 2008; Foght 2008; Rabus and Widdel 1994; Silva-Castro et al. 2013; Vieth et al. 2004). For the design of reliable and cost-efficient bioremediation methods we need to understand the controls and limitations of the biodegradation potential of natural microbial communities in aquifers (Meckenstock et al. 2015).

Petroleum hydrocarbons belong to the most abundant contaminants in aquifers (Rüegg et al. 2007; Meckenstock et al. 2004; Vieth et al. 2004; Meckenstock et al. 2010). Among them, the monoaromatic compounds benzene, toluene, ethylbenzene, and xylene (BTEX) are of major concern due to their toxicity (Bombach et al. 2009; Meckenstock and Mouttaki 2011), relatively high solubility and mobility (Chapelle 2000; Foght 2008), and broad use. BTEX compounds such as toluene have repeatedly been used as model chemicals in lab and field studies, since aerobic and anaerobic degradation pathways are known and bacterial cultures of key degraders are easily available (Meckenstock et al. 2004; Fischer et al. 2006; Mak et al. 2006; Foght 2008).

The biodegradation of BTEX in aquifers has frequently been observed. Both monitored and enhanced natural attenuation (MNA and ENA, respectively) are applied as sole remediation strategy for these compounds. Nonetheless, the ecology of the degrading microorganisms is hardly understood and thus the real biodegradation potential under in situ conditions remains unknown. Recent studies on the biodegradation of aromatic hydrocarbons in flow-through lab-studies and in the field shed some light on the limitation of biodegradation by transverse dispersive mixing (Anneser et al. 2008, 2010; Bauer et al. 2008, 2009; Eckert et al. 2015). Only if both the electron donor and a favorable electron acceptor are available, bacteria can degrade the contaminant. As a result, biodegradation activities are concentrated along the fringes of contaminant plumes at quasi-steady state (Anneser et al. 2008; Bauer et al. 2008). However, even if mixing does not control biodegradation, the interdependencies between contaminant transport, microbial transformation of the contaminants, microbial growth, and microbial transport hamper the predictability of biodegradation (Meckenstock et al. 2015).

Biodegradation coupled to bacterial growth can be simulated using analytical and numerical models. The biokinetic model parameters are commonly derived from batch experiments. Batch reactors are perfectly mixed closed systems with large water-to-solid ratios. Typically, comparably high contaminant concentrations are applied in incubation experiments. The substrate is usually the only limiting factor (Hofmann et al. 2016). In comparison, porous aquifers are open systems with small water-to-solids ratio and incompletely mixed. They are often affected by transient flow conditions and a dynamic contaminant load. While the activity of bacteria is supposed to be high and growth is fast in liquid batch systems due to the excess of the chosen electron acceptor and nutrients as well as continuous mixing, it is currently unclear how flow-through conditions, such as the flow velocity, and the sediment matrix influence biodegradation and microbial growth. The yield (i.e. the substrate carbon converted into biomass carbon) has been reported to be as high as 0.5 to 0.8 in batch and chemostat cultures dependent to the substrate applied (Ho and Payne 1979; Payne and Wiebe 1978, and references therein), while data from natural aquatic systems hint at considerably lower values (del Giorgio and Cole 1998; Hofmann and Griebler 2018). The sigmoid growth curve in batch cultures reflect exponential microbial growth followed by a plateau in cell density, mainly governed by the depletion of the substrate. In sediment flow-through systems, bacteria suspended in the mobile aqueous phase and attached to the sediment surfaces coexist and partition. In fact, in aquifers the majority of bacteria (> 99%) are usually found attached to the sediments (Griebler and Lueders 2009). Dependent on the continuous substrate load, a balance of microbial biomass between sediment and pore water is expected to establish (Griebler et al. 2002; Zhou et al. 2012). Various other factors, such as temperature, pH, availability of an energy source, quality of the substrate, toxicity, availability of terminal electron acceptors, and microbial food web interactions influence in situ microbial growth and contaminant degradation (Chapelle 2000; Meckenstock et al. 2015).

The discrepancy in conditions between flow-through and batch systems consequently raises the question how representative batch-derived rate coefficients of biodegradation and microbial growth are. Empirical findings regarding the comparability of biodegradation in batch and flow-through systems are ambiguous. While some studies reported that batch-derived biokinetic parameters adequately described biodegradation in flow-through systems (e.g., Kelly et al. 1996; Schirmer et al. 2000), others observed significant deviations (e.g., Simoni et al. 2001; Ballarini et al. 2014). In order to clarify the influence of flow conditions on biodegradation and microbial growth, we conducted a series of growth experiments using toluene as a model contaminant in batch systems and flow-through sediment microcosms applying different toluene concentration and different flow velocities with the aerobic toluene degrader *Pseudomonas putida* strain F1, the anaerobic denitrifier *Aromatoleum aromaticum* strain EbN1, and a natural microbial community from aquifer sediments. Regular measurements included the concentrations of toluene, oxygen, and cell numbers. By performing experiments in numerous replicated mini sediment columns that were successively sacrificed in the course of the experiments, we could also follow growth of the attached microbes over time. All experimental data were analyzed by reactive-transport modeling considering mobile (pore-water) and immobile (sediment) bacteria. We chose toluene as the model contaminant because well characterized toluene-degrading bacterial strains are available, but we expect that the qualitative findings of this study are applicable to the degradation of other aromatic hydrocarbons too.

## Materials and methods

### Bacteria strains and media

We used the toluene-degrading strains *Pseudomonas putida* F1 (aerobic) and *Aromatoleum aromaticum* EbN1 (denitrifying) as model organisms. Pre-cultures of both strains were grown in 100 mL serum bottles at room temperature (20 °C) in the dark with 70 µM toluene as the sole carbon and energy source. The groundwater medium was a bicarbonate-buffered freshwater medium (Widdel and Bak 1992) prepared oxic—for experiments with *P. putida*—or anoxic—for experiments with *A. aromaticum* as described elsewhere (Bauer et al. 2008, 2009). For batch experiments, we amended the respective medium with varying concentrations of toluene in closed serum bottles (100 mL) before inoculation with the bacteria. For *A. aromaticum* strain EbN1, the medium was autoclaved under N_2_ atmosphere and cooled down flushing the headspace with N_2_/CO_2_ (80:20). The medium was then transferred to serum bottles avowing oxygen penetration. Again the headspace in the serum bottles was flushed and replaced by N_2_/CO_2_ (80:20) before capped with Viton stoppers. Toluene (99.5%; Aldrich, USA) was injected with a sterile glass syringe through the Viton stoppers to obtain concentrations between 10 µM and 1 mM in the liquid phase.

In the sediment column experiments, we provided two media, one containing the electron donor (toluene) and the other the electron acceptor (oxygen or nitrate). They were mixed directly at the column inlet to avoid growth of bacteria back into the medium reservoirs (Hofmann et al. 2016). In experiments with *P. putida* F1, one medium was oxygenated while the other was anoxic but contained toluene. With *A. aromaticum* EbN1, both media were oxygen-free, one containing toluene and the other nitrate. The media were contained in gastight and inert 5 L Tedlar bags (SKC, PA, USA) without headspace and protected from light.

### Batch experiments

Both strains were inoculated at a ratio of 1:10 from pre-cultures into 100 mL serum bottles carrying 70 mL of fresh medium amended with toluene (10 µM to 1 mM) as sole carbon and energy source, either saturated with oxygen and an oxic headspace or with anoxic medium amended with nitrate (10 mM) and oxygen-free (N_2_/CO_2_) headspace. Aerobic degradation experiments were incubated at a shaker (120 rpm) to ensure replenishment of oxygen from the headspace into the medium. We conducted incubations at room temperature in the dark and regularly collected samples for the analysis of toluene (GC–MS analysis), total cell counts (OD measurements, FACS analysis), and measurement of cell size (epifluorescence microscopy). Measurements were obtained by aseptically subsampling the liquid phase with a syringe through the Viton stopper.

### Column experiments using sterile aquifer sediments

**Fig. 1:**
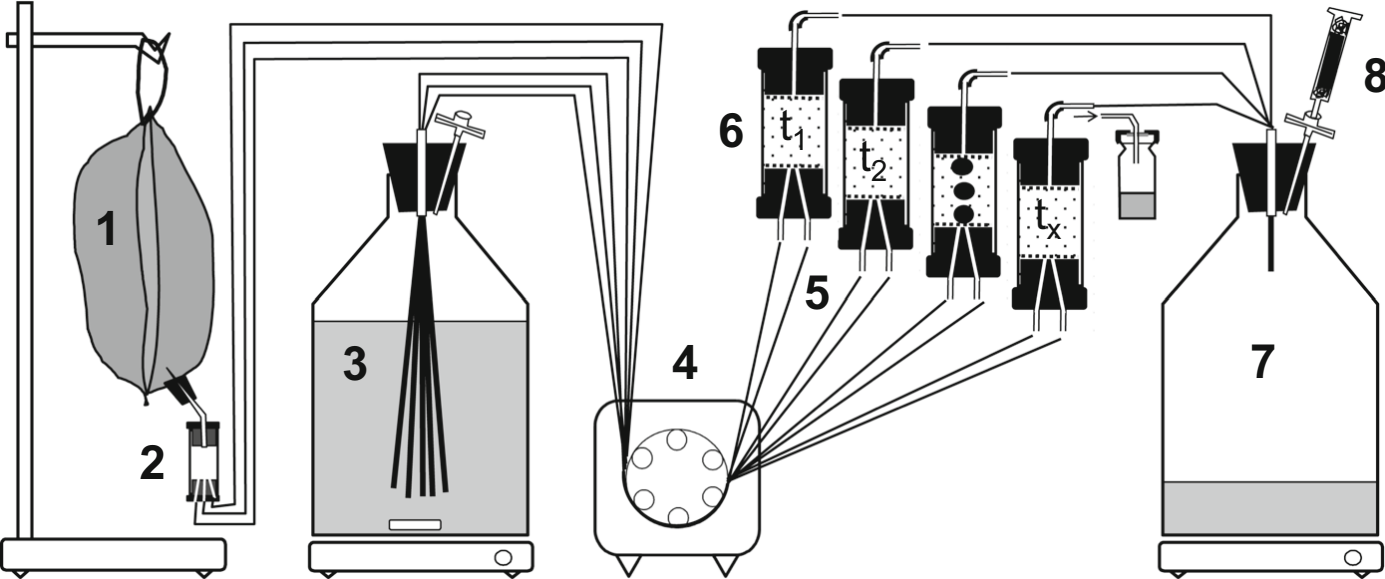
Setup of a mini sediment column experiment. (1) Gas-tight and inert Tedlar bag with anoxic medium/groundwater amended with toluene. (2) Transport of the medium via a stainless steel capillary to a splitter which feeds the capillaries/tubes that supply the individual sediment columns. (3) Oxic medium/groundwater is supplied from a reservoir bottle. (4) Multi-channel peristaltic pump. (5) Mixing of the two media come at the inlet of (6) the sediment columns. Columns are capped by Viton stoppers. At periods where there is not sampling of column outflow, the medium is transported to (7) a waste container. Mini sediment column 3 shows three spots of oxygen sensitive foil (PRESENS, Regensburg, Germany) mounted to the inner wall of the glass cylinder for non-invasive monitoring of the oxygen content in the sediment pore water (modified from Hofmann et al. 2016)

We packed mini sediment columns (material: glass, total length: 3.5 cm, active inner length: 1.6 cm, inner diameter: 1.34 cm; Fig. 1) submerged in water with sterile natural aquifer sediment with a grain size ranging from 200 to 630 µm and closed them by Viton stoppers. Packed, the columns had a sediment volume of about 2.3 mL. The in- and outflow occurred through stainless-steel capillaries in the stoppers. The flow direction was from the bottom to the top. We ran twelve columns in parallel for each treatment and maintained flow-through by means of multi-channel peristaltic pumps (Ismatech, Wertheim, Germany) using Fluran tubing. All columns carrying sterile sediment were inoculated once with the same pre-cultured strain containing a cell density of approximately 10^4^ to 10^5^ cells mL^−1^. We used 1 mL of the pre-cultured strain as inoculum in each column and left it to stand in the column for 10 min before turning on the supply of cell-free medium from the reservoir. Toluene concentrations continuously supplied to the columns ranged from 30 to 100 µM. The standard flow rate was set to 3.2 mL h^−1^. Because the porosity of the sediment was 0.3, the flow rate corresponded to a water residence time of 12.7 min and a flow velocity of approximately 1.8 m day^−1^. Overall, we tested flow rates ranging from 1 to 6.6 mL h^−1^. We collected water samples for the analysis of toluene (GC–MS analysis), total cell counts (FCM analysis), and occasional cell size measurements (epifluorescence microscopy) directly at the column inlet and outlet into small HPLC vials sealed with Teflon coated septa. The oxygen concentration within the columns was monitored by an optode technique using three spots of oxygen-sensitive foil glued to the inner wall of the glass columns. At various time points, we sacrificed columns to analyze the abundance and size of bacterial cells attached to the sediment. We determined the length and width of the cells via epifluorescence microscopy and subsequently calculated the biovolume of the cells. We divided sediments from the columns into three fractions of equal size using a sterile spatula, resulting in a bottom, a middle, and a top fraction, each representing 1/3 of the column volume (Fig. 1).

### Column experiments with active aquifer sediment

In order to compare the growth kinetics of the selected model strains to those of a natural consortium, we performed flow-through experiments in columns packed with fresh natural aquifer sediment, which we infiltrated on-line by oxygen-saturated natural groundwater. The medium containing toluene was filter-sterilized groundwater, purged anoxic with N_2_/CO_2_. The toluene concentration supplied to these columns was about 70 µM. The sampling followed the same protocols as described above.

### Chemical and microbiological analyses

Samples for toluene measurement collected at the column inlets and outlets were transferred to close GC vials (Fig. 1) containing NaOH to terminate bacterial activity. Ethylbenzene was spiked prior to analysis as internal standard. We determined concentrations of toluene via headspace analysis by GC–MS following the protocols described in Anneser et al. (2008, 2010). Concentrations of nitrate were determined by ion chromatography (Dionex AS3500, Idstein, Germany).

Bacterial cell numbers in water and sediment samples were determined by flow cytometry (FCM). For water samples, 1 mL of sample was placed into an Eppendorf tube and fixed with 100 µL of glutardialdehyde to a final concentration of 2.5%. With sediment samples, a 0.5 mL aliquot was placed in a 2 mL Eppendorf tube and fixed with 1 mL of 2.5% glutardialdehyde solution. Fixed samples were stored at 4 °C until further analysis. Later, sediment samples were further processed as described in Bayer et al. (2016). We stained water samples as well as the samples containing bacteria detached from sediment in triplicates with SybrGreen I (1000x, Molecular Probes, Invitrogen Life Sciences, 1 µl/mL) and determined cell densities in a Cytomics FC500 flow cytometer (Beckman Coulter System). The instrument settings for our experiment was: forward scatter 350 mV, sideward scatter 300–370 mV, bandpass filter 530 nm 500–580 mV and green fluorescence, bandpass filter 610 nm 650 mV and red fluorescence. The signal threshold was adjusted to 200 mV for both scatters to minimize background noise (Bayer et al. 2016).

### Modeling of batch experiments

#### Direct utilization of toluene for growth

In the standard model, we assume that the bacteria directly grow on the degradation of toluene. The electron acceptor is considered available in excess, and biomass decay is neglected. Then the standard Monod equations read as:

1 $$\frac{dX}{dt} = \mu_{max} \cdot \frac{{c_{tol} }}{{c_{tol} + K_{tol} }} \cdot X$$

2 $$\frac{{dc_{tol} }}{dt} = - \frac{1}{Y}\frac{dX}{dt}$$

in which *µ*_*max*_ [s^−1^] is the maximum specific growth rate constant, *c*_*tol*_, [µM] and *X* [cells L^−1^] are the concentration of toluene and bacteria, respectively, whereas *K*_*tol*_ [µM] and *Y* [cells/µmol] are the half-saturation concentration of toluene and the yield coefficient. This system of ordinary differential equation are subject to initial values of the two concentrations and was solved with the ode-solver ode45 of Matlab, which is an explicit Runge–Kutta solver of fourth order.

#### Consideration of a metabolite

In a second model, we assume that the bacteria first transform toluene to a metabolite without growth, and then grow on the degradation of the metabolite. A suitable candidate metabolite is methyl-catechol. The modified equations read as:

3 $$r_{tol} = r_{tol}^{max} \cdot \frac{{c_{tol} }}{{c_{tol} + K_{tol} }} \cdot X$$

4 $$r_{met} = r_{met}^{max} \cdot \frac{{c_{met} }}{{c_{met} + K_{met} }} \cdot X$$

5 $$\frac{{dc_{tol} }}{dt} = - r_{tol}$$

6 $$\frac{{dc_{met} }}{dt} = r_{tol} - r_{met}$$

7 $$\frac{dX}{dt} = Y \cdot r_{met}$$

in which [µM s^−1^] and [µM s^−1^] are the transformation rates of toluene and the metabolite, [µmol cells^−1^ s^−1^] and [µmol cells^−1^ s^−1^] are the corresponding maximum specific rates, and *c*_*met*_, [µM] is the concentration of the metabolite with the corresponding half-saturation concentration *K*_*met*_ [µM].

### Reactive-transport modeling

#### Governing equations

We simulate microbial growth in the column systems coupled to one-dimensional reactive-transport with a numerical model that considers three mobile components, namely toluene (electron donor and carbon source), oxygen (electron acceptor), and suspended bacteria as well as the attached bacteria as immobile component. We model microbial growth of attached and suspended bacteria, depending on the simultaneous presence of toluene and oxygen, by dual Monod kinetics:

8 $$r_{growth}^{att} = \mu_{max} \cdot \frac{{c_{tol} }}{{c_{tol} + K_{tol} }} \cdot \frac{{c_{ox} }}{{c_{ox} + K_{ox} }} \cdot X_{att}$$

9 $$r_{growth}^{mob} = \mu_{max} \cdot \frac{{c_{tol} }}{{c_{tol} + K_{tol} }} \cdot \frac{{c_{ox} }}{{c_{ox} + K_{ox} }} \cdot X_{mob}$$

in which *µ*_*max*_ [s^−1^] is the maximum specific growth rate constant, *c*_*tol*_, *c*_*ox*_ [µM], *X*_*att*_ [cells L_sed_^−1^] and *X*_*mob*_ [cells L^−1^] are the concentration of toluene, oxygen, attached, and mobile bacteria, respectively, whereas *K*_*tol*_ and *K*_*ox*_ [µM] are the half-saturation concentrations of toluene and oxygen, respectively. The concentration of attached cells X_att_ is expressed in number of cells per bulk volume of the sediments. Initially, we applied the same kinetic rate coefficients to the mobile and attached bacteria as expressed in Eqs. (8) and (9). However, due to the short residence time of mobile bacteria in the 1.6 cm long columns, growth of mobile bacteria was found to be insignificant, and was neglected in the further mathematical analysis.

One-dimensional transport of toluene and oxygen in the column system and their consumption due to growth of attached bacteria can be described by a system of coupled advection–dispersion-reaction equations (in which we have neglected sorption):

10 $$\frac{{\partial c_{tol} }}{\partial t} = - v\frac{{\partial c_{tol} }}{\partial x} + D\frac{{\partial^{2} c_{tol} }}{{\partial x^{2} }} - \frac{1}{Y}\left( {r_{growth}^{att} + r_{growth}^{mob} } \right)$$

11 $$\frac{{\partial c_{ox} }}{\partial t} = - v\frac{{\partial c_{ox} }}{\partial x} + D\frac{{\partial^{2} c_{ox} }}{{\partial x^{2} }} - \frac{{f_{ox} }}{Y}\left( {r_{growth}^{att} + r_{growth}^{mob} } \right)$$

with the linear transport velocity *v* [m s^−1^], the longitudinal dispersion coefficient *D* [m^2^ s^−1^], the microbial growth yield *Y* [cells µmol_tol_^−1^] and the ratio of stoichiometric coefficients of oxygen and toluene *f*_*ox*_ [µmol_tox_ µmol_tol_^−1^].

Results from the column experiments showed that the number of attached bacteria stopped increasing beyond a maximum value, indicating that there was a maximum carrying capacity of attached bacteria (*X*_*att*_^*max*^ [cells L_sed_^−1^]) in the system. However, even when *X*_*att*_^*max*^ was reached, the attached bacteria continued to replicate. In the model, the new-grown cells are released to the mobile aqueous phase and finally flushed out of the column. This release of new-grown cells from the sediment surface to the mobile aqueous phase has already been observed in earlier studies on microbial transport under growth conditions (e.g., Clement et al. 1997; Murphy et al. 1997; Yolcubal et al. 2002; Jordan et al. 2004; Mellage et al. 2015). We accounted for this process in the model by the dynamic detachment rate *r*_*daughter*_ [cells L_sed_^−1^ s^−1^]:

12 $$r_{daughter} = r_{growth}^{att} \cdot \frac{{X_{att} }}{{X_{att}^{max} }}$$

If *X*_*att*_^*max*^ is not yet reached, new-grown cells partially stay attached and partially are released to the mobile aqueous phase. When the carrying capacity is approached, the term *X*_*att*_/*X*_*att*_^*max*^ approaches unity and all new-grown cells are released to the aqueous phase.

Attachment of suspended bacteria to the sediment surface is described by the modified first-order attachment rate *r*_*attach*_ [cells L^−1^ s^−1^]:

13 $$r_{attach} = k_{att} \cdot X_{mob} \cdot \left( {1 - \frac{{X_{att} }}{{X_{att}^{max} }}} \right)$$

in which *k*_*att*_ [s^−1^] is the first-order attachment rate coefficient and the term $\left( {1 - \frac{{X_{att} }}{{X_{att}^{max} }}} \right)$ is introduced to account for the carrying capacity (Ding 2010). The rate of change of attached *X*_*att*_ [cells mL_sed_^−1^] and mobile *X*_*mob*_ [cells mL^−1^] bacteria is described by:

14 $$\frac{{\partial X_{att} }}{\partial t} = r_{growth}^{att} + n \cdot r_{attach} - r_{daughter}$$

15 $$\frac{{\partial X_{mob} }}{\partial t} = - v\frac{{\partial X_{mob} }}{\partial x} + D\frac{{\partial^{2} X_{mob} }}{{\partial x^{2} }} - r_{attach} + \frac{1}{n}r_{daughter}$$

Note that the carrying capacity *X*_*att*_^*max*^ is a prescribed model parameter that needs to be obtained by fitting the model to data. The model itself does not explain the mechanisms determining the carrying capacity.

#### Numerical methods

We discretized the coupled system of one-dimensional reactive-transport equations in space by the cell-centered Finite Volume Method with a spatial discretization of ∆*x* = 0.5 mm. We applied upwind differentiation of the advective term and set the dispersion coefficient to 1.95 × 10^−8^ m^2^ s^−1^. The coupled system of spatially discretized reactive-transport equations was integrated in time by an implicit Euler method with adaptive time stepping and a maximum time-step size of 600 s. The resulting system of coupled non-linear algebraic equations was linearized by the Newton–Raphson method, and the UMFPACK solver implemented in Matlab was used to solve the resulting system of linear equations. The code was written as a Matlab program.

## Results

### Batch experiments

**Fig. 2:**
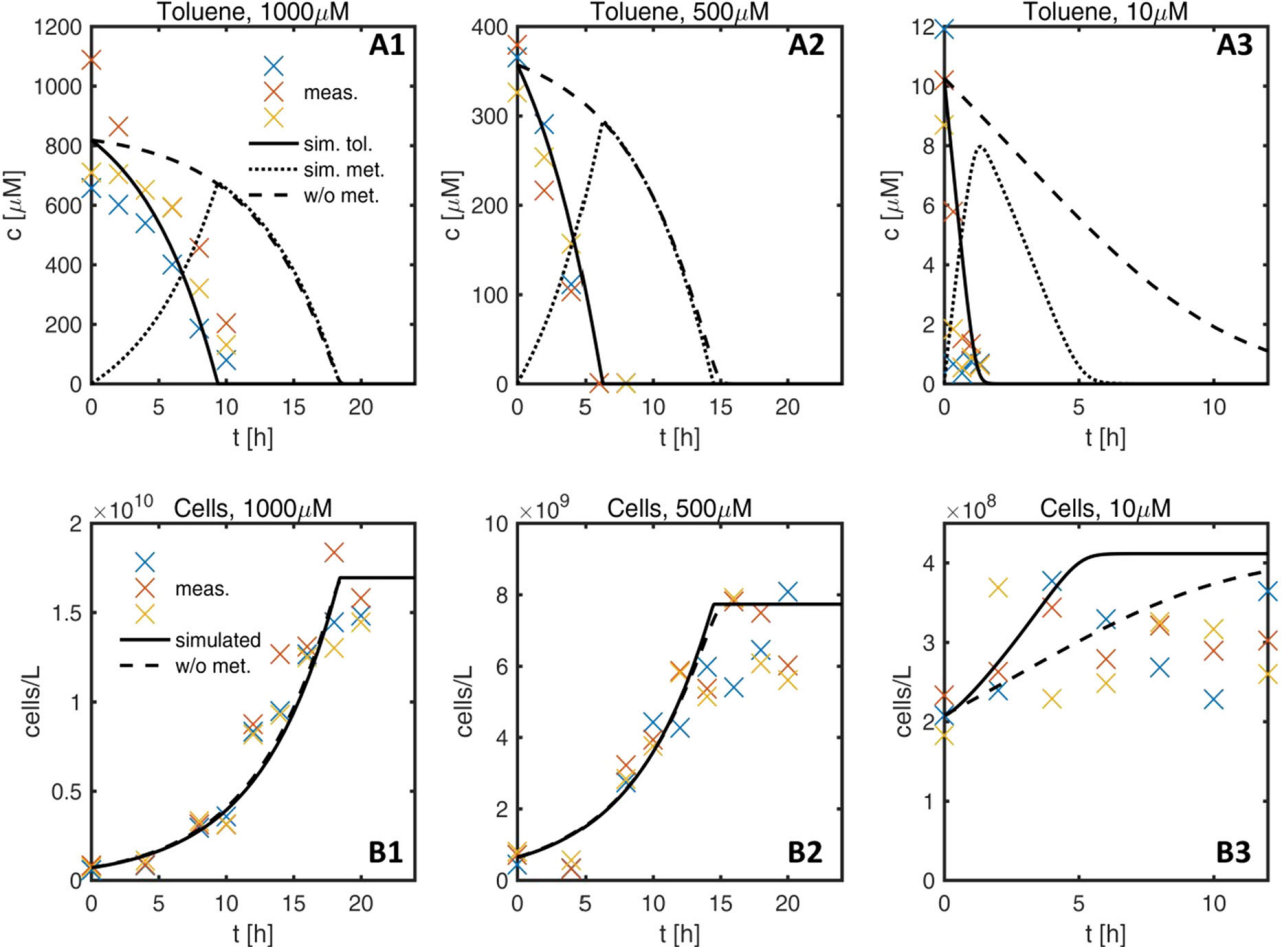
Measured and simulated toluene concentrations and bacterial growth of *P. putida* F1 over the course of batch experiments with different substrate starting concentrations. The color coded data points represent measured values of toluene and cells/ml, respectively, from batch triplicates. Two scenarios were considered, (1) with the formation of the central metabolite 3-methyl catechol (solid back lines—simulated toluene, dotted back lines—simulated metabolite), and (2) without metabolite (black dashed lines)

Figure 2 shows measured and simulated concentrations of toluene and cell numbers in the batch experiments of aerobic toluene degradation with *Pseudomonas putida* F1. The lines show fitted model results, where the dashed lines represent the standard model, in which a given fraction of toluene is immediately used for biomass growth, and the solid lines represent the model with an intermediate metabolite (shown as dotted line) that can be further utilized for assimilation. It is obvious that the standard model fails at reproducing the data because the decrease in toluene concentrations precedes the increase in cell numbers. In the standard model, the fitted maximum specific growth rate *µ*_*max*_ is 4.25 ± 0.24 day^−1^ and the fitted Monod constant *K*_*tol*_ is 10.9 ± 2.83 µM, with a yield coefficient of *Y* = 2.83 × 10^8^ cells µmol_tol_^−1^. In the model with the metabolite, the first reaction is considerably faster than the second one (*r*_*tol*_^*max*^/*Y* = 24.06 ± 0.01/*d* vs. *r*_*tol*_^*max*^/*Y* = 4.19 ± 0.03/*d*, in which the scaling with the yield is chosen to make the numbers comparable to *µ*_*max*_ of the standard model), and the Monod constant *K*_*tol*_ of 1.45 ± 0.002 µM is much better constrained.

In Figure S1 (Supporting Information) we show that the substrate carbon converted to biomass carbon over the entire experiment increased with the initial toluene concentration but plateaued for initial toluene concentrations of about 150 µM and higher. At the end of all experiments, toluene was completely degraded. The finding of a maximum biomass concentration indicates decreasing carbon-assimilation efficiency with increasing carbon and electron-donor supply.

To fit the toluene and cell data for the batch experiments under nitrate-reducing conditions with *Aromatoleum aromaticum* EbN1, it was not necessary to consider a metabolite (see the model fit assuming direct utilization of toluene for growth in Figure S2 of the Supporting Information). The fit of the classical Monod-growth-model to all toluene and cell data revealed a *µ*_*max*_ of 0.35 day^−1^, *K*_*tol*_ = 21.7 µM, and *Y* = 1.38 × 10^8^ cells µmol_tol_^−1^. The maximum specific growth rate of the aerobe *P. putida* F1 was about 10 times higher than that of the denitrifier *A. aromaticum* EbN1, and *K*_*tol*_ was 15 times larger for the denitrifyer.

### Sediment column experiments

We performed experiments with different toluene concentrations in the inflow and different flow velocities with *P. putida* F1, *A. aromaticum* EbN1, and a natural aquifer microbial community. Table 1 summarizes key results of the individual experiments with regard to toluene degradation, oxygen consumption, and microbial growth.

**Table 1:** Comparison of column experiments with *P. putida* F1, *A. aromaticum* EbN1, and a natural toluene degrading community at different flow rates and toluene source concentrations

Exp	$C_{Tol}^{in}$ (µM)	*v* (m/day)	ΔTol (µM)	ΔTol (%)	ΔO_2_ (µM)	*f*_*ox*_ ($\upmu {\text{mol}}_{{{\text{O}}_{ 2} }} /\upmu {\text{mol}}_{\text{Tol}}$)	New cells (× 10^8^ cells)	Cells flushed out (%)	Yield (× 10^7^ cells/µmol _Tol_)	Cells attached (%)
*P. putida F1*										
A	70 (66.9)	0.6	66.9	100	207	3.1	4.4	70	3.6	99
B	70 (65)	1.8	59.6	92	203	3.4	14	76	4.0	99
C	70 (73.5)	3.6	51	69	261	5.1	28	79	5.3	99
D	30 (26.3)	1.8	26.3	100	104	3.95	6.6	72	5.8	99
E	100 (113)	1.8	67.2	59	247	3.7	53	93	11.7	98
*A. aromaticum EbN1*										
F	70 (85.4)	1.8	63.1	73	–	–	10	20	3.0	99
Natural microbial community										
G	70 (73.1)	1.8	73.1	100	256	3.5	11	50	3.5	99

*C*_*Tol*_^*in*^: toluene concentration in the inflow (target concentrations are listed first, actual concentrations given in brackets), *v*: velocity, ΔTol: difference in toluene concentration between in- and outflow, ΔO_2_: difference in oxygen concentration between in- and outflow, *f*_*ox*_: stoichiometric ratio between oxygen and toluene, New Cells: increase in cell numbers

#### Toluene degradation

**Fig. 3:**
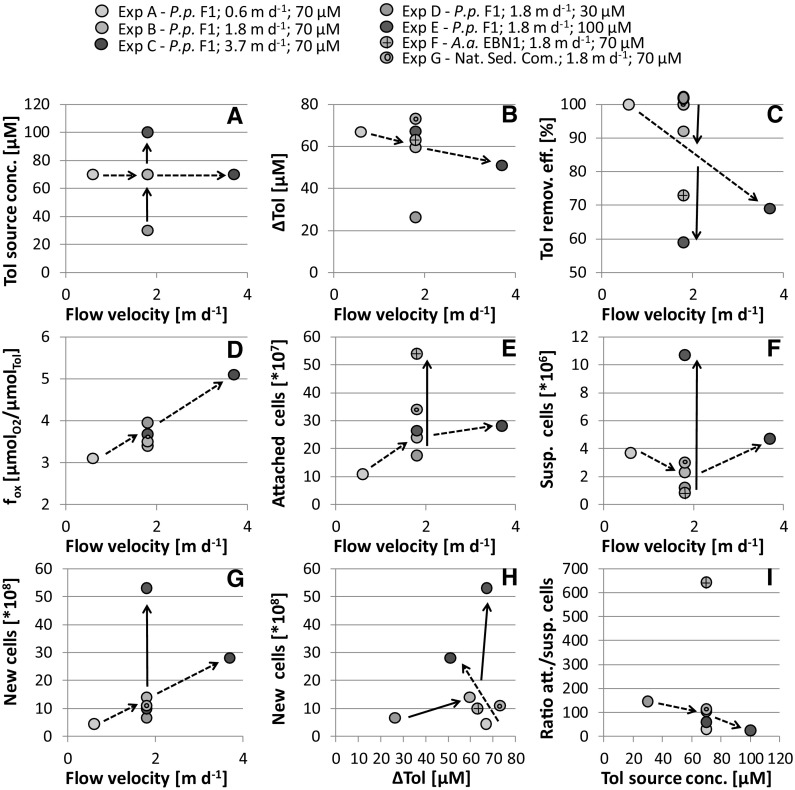
Influence of toluene inlet concentration (solid arrows) and flow velocity (dashed arrows) on biodegradation, growth and yield, as well as the distribution of bacterial cells on the sediment and in porewater

**Fig. 4:**
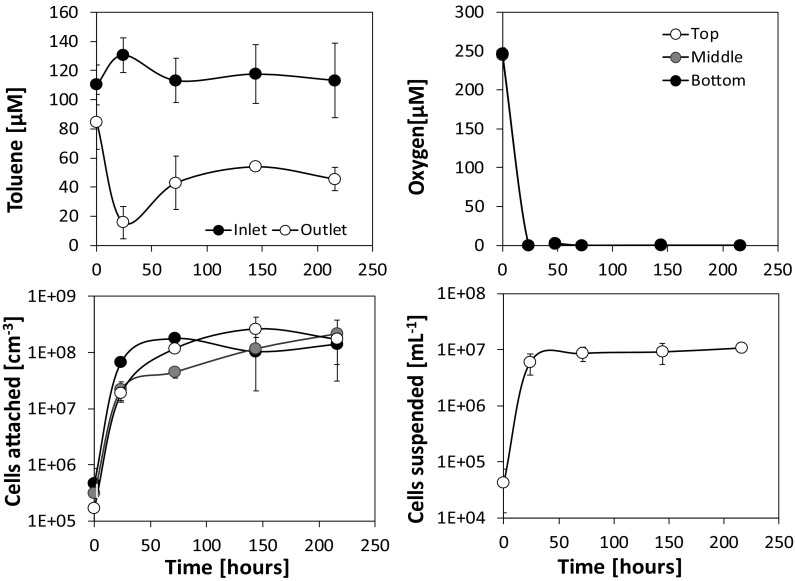
Aerobic toluene degradation and growth of *P. putida* F1 at a continuous source concentration of 100 µM and a flow velocity of 1.8 m day^−1^ (Exp E). Values are means of triplicate measurements ± SD

The reduction in toluene concentration (∆Tol) in the individual column experiments with *P. putida* F1 (Exp A to Exp C) showed a linear decrease with increasing flow velocity at identical inflow concentration (Table 1, Fig. 3b, c). The total toluene transformation within 192 h ranged from 12.2 µmol in Exp A (v = 0.6 m day^−1^) to 53.6 µmol in Exp C (v = 3.6 m day^−1^) exhibiting a positive trend, i.e. an increase with increasing flow velocity and thus increasing toluene mass flux. With respect to the toluene removal efficiency, the experiment with the lowest flow velocity, Exp A, showed 100% toluene removal followed by Exp B and C. At the highest flow velocity, only 69% of the toluene could be degraded aerobically by *P. putida* (Table 1). With regard to the inflow concentration, ∆Tol increased with increasing C_Tol_. However, at the highest toluene concentration in the inflow (100 µM; Exp E) the data clearly hint at an oxygen limitation and degradation efficiency dropped to 59% (Fig. 4; Table 1). After establishment of full biodegradation activity, also experiments D (*P. putida* at lower source concentration) and G (natural aerobic consortium) revealed 100% toluene removal efficiency (Table 1; Fig. 3c). Further relationships between toluene source concentration, flow velocity, and biodegradation efficiency are depicted in Fig. 3a–c.

Figure 4 exemplarily depicts the time series of concentrations and cell numbers for one set of experimental conditions (Exp. E: *P. putida*, *c*_*tol*_ = 100 µM, *v* = 1.8 m day^−1^). As can be seen, the concentration of toluene in the column outflow as well as the oxygen concentration within the sediment column immediately started to decline and both leveled off after 1–2 days at concentrations of approximately 45 µM toluene and < 0.2 µM dissolved oxygen, respectively (Fig. 4). No differences in the oxygen values were found between the bottom, middle, and top column observation points indicating that oxygen was readily consumed in the bottom (inflow) part of the column. Experiments at lower inflow concentrations of toluene and varying flow velocities exhibited similar patterns (see Supplementary Information, Figs. S3–S6).

In the column experiment F, which is similar to that depicted in Fig. 4, toluene degradation was examined under nitrate reducing conditions by the strain *A. aromaticum* EbN1. Here, the bacterial population took 3 days to establish the full toluene degradation capacity, which was considerably slower than the aerobic culture *P. putida*. We chose a nitrate concentration of 500 µM because this concentration would be sufficient for the complete transformation of the foreseen 70 µM toluene in a perfectly mixed solution. Unfortunately, the actual inflow concentration of toluene was about 80 µM and thus a bit higher than intended. In fact, the columns outflow still contained about 20 µM of toluene, pointing at a nitrate limitation under the flow-through conditions in the mini sediment column (Fig. S7).

The natural microbial sediment community fed by oxic groundwater containing 70 µM toluene (Exp. G) was able to readily degrade toluene, albeit the maximum degradation efficiency was reached much later than with the specific degrader strains applied, i.e. after 6 days compared to 1–2 days with the *P. putida* F1 and 3 days with *A. aromaticum* EbN1. After establishment of quasi steady-state conditions in the sediment columns, the change of toluene concentration between the in- and outlet under similar experimental conditions (*c*_*tol*_ = 70 µM, *v* = 1.8 m day^−1^) by *P. putida* F1 (59.6 µM), *A. aromaticum* EbN1 (63.1 µM) and the natural microbial community (73.1 µM) were in a similar range.

Based on toluene and oxygen measurements (∆O_2_ and ∆Tol at fully established biodegradation activity; Table 1) conducted for the column experiments with *P. putida* F1 and the natural aquifer community, we calculated the empirical stoichiometric ratio *f*_*ox*_ between oxygen consumption and toluene degradation, as well as bacterial growth. The *f*_*ox*_-values obtained for the different experiments fell in the range of 3.1–5.1 $\upmu {\text{M}}_{\text{Tol}} \;\upmu {\text{M}}_{{{\text{O}}_{ 2} }}^{ - 1}$. We found the highest stoichiometric ratio with the highest flow velocity (Exp C) and thus the highest toluene mass flux. *f*_*ox*_ decreased together with the flow velocity (Fig. 3d). No pronounced differences in *f*_*ox*_ nor a consistent trend were observed with varying inflow concentration (Fig. 3d). In the experiment with the natural aquifer community a similar *f*_*ox*_-value (3.5) was found as with *P. putida* F1 under comparable environmental conditions (Table 1).

A high stoichiometric ratio *f*_*ox*_ translates into low carbon biomass yield. The highest yield under quasi steady-state conditions was thus with the lowest toluene mass flux. However, dissecting the column experiments into an initial phase of rapid cell production and the subsequent phase at quasi-steady state revealed a very dynamic biomass yield. In almost all experiments, carbon assimilation was extremely high in the initial phase of growth and then decreased within two, maximal 3 days to a lower constant value when the carrying capacity for the cell density was reached (see the carbon assimilation efficiency in Fig. S8).

Due to technical problems, nitrate concentrations could not be measured in the column experiment F with *A. aromaticum* EbN1. Therefore, we could not evaluate the stoichiometry between toluene degradation and nitrate consumption under nitrate-reducing conditions.

#### Microbial growth

In all column experiments the number of bacterial cells attached to the sediment substantially increased within the first 1–3 days. As exemplarily depicted in Fig. 4, the number of attached cells per volume sediment, here at an inflow concentration of 100 µM and a flow velocity of 1.8 m day^−1^, increased by more than three orders of magnitude within 72 h and then stayed rather constant for the remaining time of the experiment. The maximum density of attached cells reached 2.6 × 10^8^ ± 1.6 × 10^8^ cells mL_sed_^−1^. The number of cells suspended in the pore-water collected at the column outflow also increased by two orders of magnitude reaching a constant value of about 1 × 10^7^ cells mL^−1^ already after 48 h. This constant outwash of 1 × 10^7^ cells mL^−1^ following day two, pointed at an actively growing attached bacterial population, releasing its daughter cells into the mobile water phase. Since 99% of the cells per sediment volume were found attached to the sediment surface (see Table 1) and the water residence time in the columns was considerably short (12.7 min), toluene degradation and microbial growth could be fully attributed to the attached populations in the column system. However, over the entire phase of the experiment (192 h), 97% of all newly produced cells were transported out the columns in experiment E.

We observed similar patterns for the other column experiments (Table 1). For *P. putida* F1, the final number of cells associated to the sediment showed a positive trend with increasing flow velocity as well as with increasing toluene inlet concentration (Fig. 3e). The highest abundance of attached cells was obtained in the experiments with the denitrifyer *A. aromaticum* (Fig. 3e). In the pore-water, patterns of cell numbers were less clear. While, increasing toluene concentrations in the inlet pushed the cell numbers in the pore-water from 1.2 × 10^6^ cells mL^−1^ in Exp D (30 µM) to 1 × 10^7^ cells mL^−1^ in Exp E (100 µM), we could not observe a conclusive dependence of cell-numbers in the pore water and flow velocity (Fig. 3f). The ratio of attached to suspended cells ranged from 14 to 643, with the highest ratio for the denitrifyer *A. aromaticum* (Fig. 3i). For the aerobic strain *P. putida* F1 and with the natural aerobic consortium, we observed a lower ratio of attached to suspended cells (Fig. 3i).

Similar to the maximum total cell numbers observed in the pore-water and attached to the sediment, the number of newly grown cells, as determined by direct cell counts, systematically increased with the increase of toluene concentration in the inflow as well as with the flow velocity (Table 1; Fig. 3g, h). Consequently, we observed a similar trend for the microbial growth yield *Y* [cells µmol_tol_^−1^].

The estimated growth yield for the column experiments with *A. aromaticum* EbN1 under denitrifying conditions and the natural community, with values of 3.0 × 10^7^ and 3.5 × 10^7^ cells µmol_tol_^−1^, respectively, were in a similar range as for *P. putida* F1 (of 4.0 × 10^7^) under similar experimental conditions (70 µM toluene, *v* = 1.8 m day^−1^) (Table 2 and Fig. 3g, h).

**Table 2:** Model parameters

Parameter	Exp A	Exp B	Exp C	Exp F	Exp G
*µ*_*max*_ (1 day^−1^)	4.5	4.5	3.0	2.0	0.5
*K*_*tol*_ (µmol L^−1^)	1.1	1.1	1.1	21.7	1.1
*K*_*ox*_ (µmol L^−1^)	10^a^	10^a^	10^a^	–	10^a^
*f*_*ox*_ ($\upmu {\text{mol}}_{{{\text{O}}_{ 2} }} /\upmu {\text{mol}}_{\text{Tol}}$)	2.6	4.0	4.9	–	3.7
*k*_*att*_ (1 day^−1^)	50	50	50	50	50
(cells mL_Sed_^−1^)	0.9 × 10^8^	2.0 × 10^8^	2.7 × 10^8^	5.4 × 10^8^	3.4 × 10^8^
*Y* (cells µmol_Tol_^−1^)	3.6 × 10^7^	4.0 × 10^7^	5.3 × 10^7^	3.0 × 10^8^	3.5 × 10^7^

Parameters given in bold were fitting parameters. The maximum number of attached cells *X*_*att*_^*max*^ was set to be the highest number of cells/ml obtained from sediment analysis in each experiment. The yield coefficient Y in the model was calculated from the column data by comparing the number of newly formed cells to the amount of degraded toluene. K_tol_ was taken from the preceding batch experiments, and K_ox_ was obtained from the literature

^a^Bauer et al. (2009)

Even though a large fraction of the newly grown cells in the flow-through experiments conducted with *P. putida* F1 were flushed out of the column over time (60–93%), at any given time point the majority of cells per volume of water-saturated sediment was found to be attached to the sediment surface, regardless of the experiment (≥ 98%, Table 1). The experiment using the natural aquifer community showed a considerably lower percentage of washed-out cells (50%), and the experiment using *A. aromaticum* EbN1 had the lowest percentage of washed-out cells (20%, Table 1). While the measurements of attached cells showed a distinct gradient in cell numbers along the length of the column in Exp B, with the highest cell numbers in the bottom (inlet) part of the column, no pronounced spatial gradient along the bottom, middle, and top parts of the columns was found in the other experiments (Figs. S3–S8).

We estimated the growth yield in our experiments in two ways: First, by converting the *f*_*ox*_-values (see above) to yield coefficients, and second by comparing the amount of newly grown cells to the toluene mass degraded. The range of *f*_*ox*_-values between 2.6 and 5.5 $\upmu {\text{mol}}_{\text{Tol}} \; \upmu {\text{mol}}_{{{\text{O}}_{ 2} }}^{ - 1}$ (Table 1), indicates carbon assimilation efficiencies of 0.39–0.72. Summing up all newly formed cells within the individual experiments led to similar carbon assimilation efficiencies of 0.35–0.7. The bacterial cell size of *P. putida* F1, sporadically determined via fluorescence microscopy, was found to be rather constant in the column experiments, with an average length of 1.6 µm and an average width of 0.8 µm. This corresponds to a biovolume of roughly 0.5 µm^3^ per cell. Consequently, a mean cell carbon content of 130 fg was used for the conversion of cell numbers into cell carbon.

#### Reactive-transport modeling

**Fig. 5:**
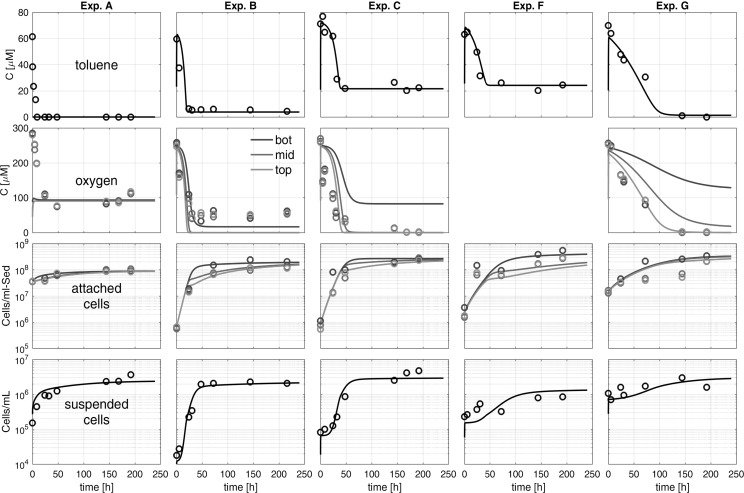
Comparison of the experimental data and simulation results for selected column experiments; *bot* column bottom (close to inlet), *mid* middle part of the column, *top* top part of the column (close to outlet)

Figure 5 depicts concentration and cell-count time series of the column experiments A–C, performed with *P. putida* F1 at different flow velocities, as well as the experiment performed with the denitrifier *A. aromaticum* EbN1 (Exp F) and the natural microbial community (Exp G), together with the corresponding time series obtained by reactive-transport simulations. Table 2 lists the individual model parameters. The maximum specific growth rate *µ*_*max*_, the stoichiometric coefficient for oxygen consumption *f*_*ox*_, and the attachment rate coefficient *k*_*att*_ were fitting parameters. All remaining parameters were either calculated from the batch and column data prior to the simulations or taken from the literature.

The model reproduces the observed breakthrough curves of toluene and suspended cells at the column outlet, as well as the temporal evolution of the number of attached cells in the bottom, middle, and top parts of the columns very well, with exception of dissolved oxygen. The largest discrepancy between experimental and simulation results was found for the experiment with the natural microbial community (Exp G), for which the measurements indicated complete oxygen depletion already in the bottom (inflow) part of the column, whereas the simulation results showed a continuous gradient in oxygen concentrations along the length of the column.

While the concentration of suspended cells in the outflowing water was in the same range in all experiments conducted with *P. putida* F1 at a toluene concentration of 70 µM, the flux of cells leaving the column was increasing with higher flow rates. The leveling off in the number of attached cells at a maximum value is enforced in the model by introduction of the maximum number of attached cells *X*_*att*_^*max*^ in Eqs. (12) and (13). Without this term, we would have achieved higher biomass concentrations, particularly close to the column inlet. The model also captures the continuous outflow of 2 to 4 × 10^6^ cells mL^−1^ in all experiments conducted with *P. putida* F1 and 70 µM toluene well because it simulates the release of new-grown cells from the sediment (immobile phase) to the mobile aqueous phase by the dynamic growth-depended detachment rate *r*_*daughter*_ (Eq. 12). This term balances biomass growth once the carrying capacity of the system for attached cells is reached.

Because the vast majority of bacteria, and hence biodegradation activity per volume water saturated sediment, was located at the sediment surfaces rather than in the aqueous phase (see Table 1), toluene degradation in the columns could be almost exclusively attributed to the attached bacterial populations. Moreover, additional simulations, in which growth of suspended bacteria was accounted for, revealed the same results as simulations in which growth of suspended bacteria was neglected (data not shown). The concentration profiles of toluene and attached cells could even be reproduced without explicit consideration of mobile cells, if a logistic growth-term of the form $\left( {1 - \frac{{X_{att} }}{{X_{att}^{max} }}} \right)$ was applied to the microbial growth rate, but not to the rates of toluene and oxygen consumption. While suspended bacteria were found to be unimportant for toluene degradation, between 20% (Exp F) and 93% (Exp E) of the newly grown cells were flushed out of the columns over time (Table 1).

The remaining toluene concentration at the outlet of the columns inoculated with *P. putida* F1 increased with the flow rate from Exp B. (1.8 m day^−1^) to Exp C (3.6 m day^−1^), and the initial drop in toluene concentration was faster for Exp B than for Exp C. This is also captured by the fitted values of *µ*_*max*_ (Table 2). While the same value was applicable for Exp B and the batch experiments, a slightly lower one had to be chosen to match the results of Exp. C, which is the experiment with the higher flow velocity.

The fitted maximum specific growth rate *µ*_*max*_ of 0.5 day^−1^ in the experiment with the natural microbial community is almost one order of magnitude smaller than the value estimated for the experiment with *P. putida* F1 at the same flow velocity (*µ*_*max*_ = 4.5 day^−1^). Even though the initial abundance of attached cells was about one order of magnitude higher for the experiment with the natural community, as compared to the experiments with *P. putida* F1, the initial drop in toluene degradation was much slower. In this context, it may be worth noting that the simulation was performed under the assumption that all attached cells in the experiment with the natural community were able to readily degrade toluene, which is unlikely for a natural microbial community that consists not only of specific toluene degraders such as *P. putida* F1 (see discussion). Additional simulations, in which we assumed that only a fraction of 1% of the detected number of attached cells was able to readily degrade toluene, however, showed hardly any difference at late times because with the given maximum specific growth rate *µ*_*max*_ the necessary 100-fold increase of biomass at early times only takes 4–7 h.

The fitted maximum specific growth rate *µ*_*max*_ of 2 day^−1^ for the experiment with *A. aromaticum* EbN1 (Exp F) is almost seven times larger than the value obtained from analyzing the batch experiments. Apparently some conditions within the flow-through system provided a better environment for growth of the denitrifying culture than in the batch system, but we don’t know the decisive factor.

While the model included attachment of mobile biomass (Eq. 13), the attachment rate coefficient *k*_*att*_ was not a very sensitive parameter. Independent of the flow velocity, all experiments could be reproduced with a value of 50 day^−1^. Additional model runs, in which *k*_*att*_ was set to zero, indicated that the observed increase in the number of attached cells in the middle and top part of the column can be explained to a large extent by microbial growth during the initial phase of the experiment, when oxygen was still available along the entire column.

**Fig. 6:**
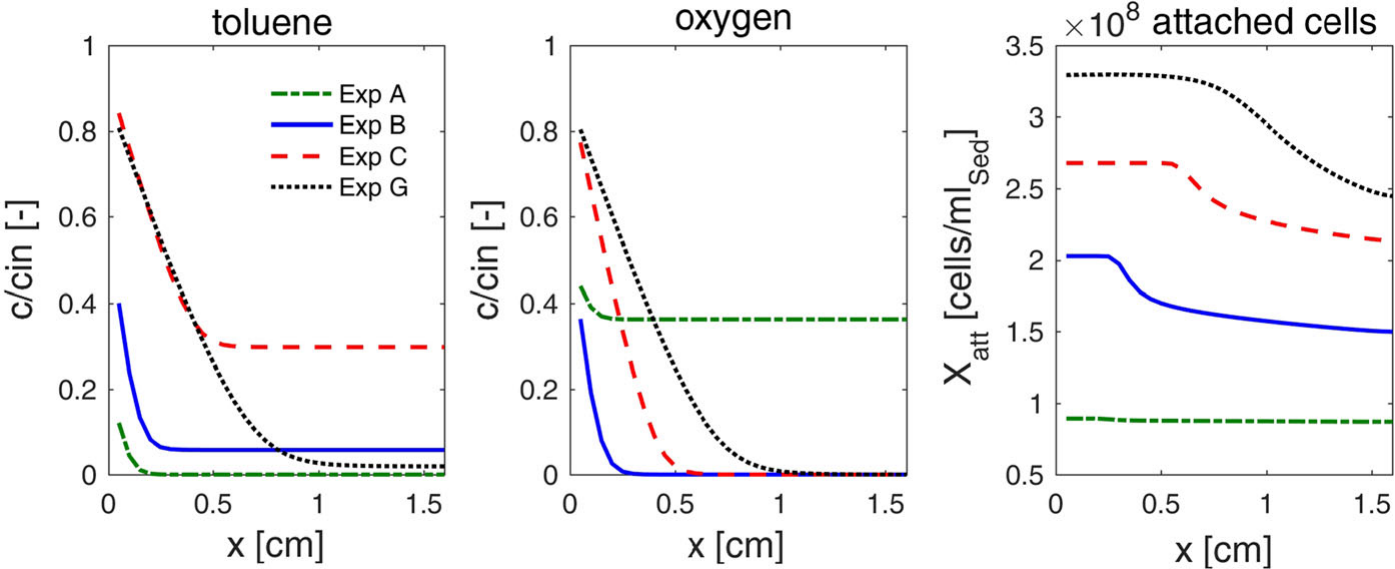
Simulated spatial profiles for toluene, oxygen and attached cell concentrations for Exp B–C, and G after steady state has been reached (t = 200 h)

Figure 6 depicts simulated spatial profiles of toluene, oxygen, and attached cells in the sediment columns for the experiments A–C (*P. putida* F1 with *C*_*Tol*_^*in*^ = 70 μM), and experiment G, conducted with the natural microbial community, after steady state has been reached (*t* = 200 h). The model predicts a rapid decrease in toluene concentrations with travel distance, and the complete consumption of oxygen within the first centimeter for all experiments. This matches the fact that no differences in oxygen profiles could be observed between the three locations in the columns.

## Discussion

### Batch versus flow-through experiments

Microbial growth observed in batch tests using different initial toluene concentrations exhibited clear first-order degradation kinetics. Model fits of the batch tests showed a maximum specific growth rate *µ*_*max*_ of 4.5 day^−1^ for *P. putida* F1, which is somewhat smaller than literature values ranging from 12 to 21 day^−1^ (Abuhamed et al. 2004; Alagappan and Cowan 2004; Reardon et al. 2000). In the batch experiments with *P. putida* F1 at higher concentrations, we observed that biomass growth continued even after toluene had already completely been consumed (see Fig. 2). In these instances the standard Monod growth model could not simultaneously explain the toluene and cell data. Yu et al. (2001) observed a similar pattern and provided evidence that the buildup and further breakdown of 3-methylcatechol, a central intermediate in toluene degradation by *P. putida* F1, was responsible for the observed mismatch. Considering a central intermediate, the chemical nature of which has not been identified in the current experiments, we could fit the batch experiments involving *P. putida* much better (see solid lines in Fig. 2). For *A. aromaticum* EbN1, the denitrifying strain, ten times lower maximum specific growth rates were found with *µ*_*max*_ values of 0.3 day^−1^. Here, literature values range between 2.2 and 2.6 day^−1^ (Evans et al. 1991; Jorgensen et al. 1995).

While the maximum specific growth rates of *P. putida* F1 in the batch and flow-through systems were similar, the mean growth rates differed. In batch systems with initial concentrations of 50 µM toluene or more, the mean growth rates were consistently higher than in column experiments with inflow concentrations of 70 µM of toluene. However, in batch systems the bacteria are exposed to a limited mass of toluene, they grow quickly, consume the toluene and stop growing because they run out of substrate. In the flow-through systems, by contrast, the bacteria exhibit a permanent supply of the substrate. They grow until they approach the carrying capacity, when they start to release the new grown cells. That is, the control of biomass growth considerably differs among the two systems.

The picture was different for the denitrifying strain *A. aromaticum* EbN1. Here the growth rates were much higher (factor of 20) in the flow-through experiments. The reason for that is not fully clear. However, there are strong indications from the sediment-column experiments that *A. aromaticum* EbN1 prefers to grow attached to surfaces as seen from the ratio between attached versus suspended cells (Fig. 3i) and the very different growth rates of suspended and attached cells in the sediment columns (Fig. S10). Without sediments to attach to, *A. aromaticum* EbN1 obviously experiences less favorable growth conditions.

In terms of yield, *P. putida* F1 was more efficient converting toluene carbon into biomass carbon at lower concentrations in the flow-through sediment columns than in the batch systems. At an initial toluene concentration of 10 µM no significant increase in cell numbers could be detected in the batch experiment. As a consequence, at low substrate concentrations, substrate turnover fuels cell maintenance and energy production rather than growth (Egli 2010). At concentrations > 100 µM, carbon assimilation efficiencies became pronounced with values between 0.38 and 0.55 in the batch experiments (Figs. S1 and S2). The latter values are well comparable to the flow-through systems (0.43–0.7).

The yield of *A. aromaticum* EbN1 was comparable to *P. putida* in the batch system. Since there was only one column experiment with *A. aromaticum* EbN1 and we lacked nitrate data for the columns, a direct comparison between batch and flow-through sediment microcosms was not valid.

### Column experiments: impact of flow velocity and inflow concentration

#### Toluene degradation and bacterial growth

In all column experiments, the difference between the inlet and outlet concentrations of toluene (ΔTol) initially increased and then reached a steady value, which we denote the maximum degradation efficiency. While steady overall turnover was eventually reached in all column experiments, the time needed to approach the steady value differed between the bacteria involved. *Pseudomonas putida* F1 generally reached maximum degradation efficiency within 1–2 days, *A. aromaticum* EbN1 needed 3 days, and the natural microbial community was the slowest with a maximum degradation efficiency approached after 6 days. We may explain this by biodegradation being related to the amount of active biomass (specific degrader) and the growth rate being related to the energy gained. In the batch experiments, the denitrifying strain *A. aromaticum* EbN1 had a much smaller maximum specific growth rate *µ*_*max*_ than the aerobic strain *P. putida* F1. The difference was smaller in the case of the column experiments. As depicted in Table 2 and Fig. S10, proliferation of attached *A. aromaticum* EbN1 cells is in the same range as that of *P. putida* F1 (see below). The natural microbial community, by contrast to the specific cultures, consisted of probably thousands of different strains, with only a small fraction readily capable of aerobic toluene degradation and related growth (Castillo and Ramos 2007; Okpokwasili and Nweke 2005; Vecht et al. 1988).

While the total amount of degraded toluene increased with the flow velocity, toluene removal efficiency, i.e. the percentage of the injected toluene mass which was degraded while being transported through the column, decreased. A similar pattern was observed for the maximum specific growth rate µ_max_ (see Tables 1 and 2, Fig. 2). An explanation is that the mass-transfer of toluene and oxygen/nitrate from the pore-water to the interior of attached bacteria exerts a stronger control on reactive turnover at higher flow velocities because the characteristic time scale of mass-transfer into the cells is independent of velocity whereas the characteristic time of advection is inversely proportional to velocity. Mass transfer limitations were put forward as explanation for decreased degradation rates in porous media by numerous studies (e.g., Dykaar and Kitanidis 1996; Simoni et al. 2001; Hesse et al. 2009). At a fixed flow velocity, increasing the toluene concentration in the inflow revealed an increase in total toluene mass degraded per unit time. The toluene removal efficiency per unit volume, on the other hand, declined with increasing concentration in the inflow (see Table 1). This is actually expected for inflow concentrations *C*_*Tol*_^*in*^ that are considerably larger than the half-saturation concentration *K*_*tol*_ of toluene. In this concentration range, the turnover hardly increases with increasing concentration, so that the pseudo first-order coefficient $\frac{1}{Y} \cdot \frac{{\mu_{max} }}{{c_{tol} + K_{tol} }}$ decreases.

We estimated the yield by two independent approaches leading to comparable results. In the first approach, we compared the amount of oxygen consumed per toluene degraded, *f*_*ox*_. For the complete mineralization of toluene to carbon dioxide and water, a value of 9 ${\text{mol}}_{{{\text{O}}_{ 2} }} \;{\text{mol}}_{\text{Tol}}^{ - 1}$ is applied, which does not account for cell maintenance, carbon assimilation, and growth. Under growth conditions, however, bacteria oxidize only a part of the substrate to carbon dioxide to gain energy, while they assimilate the other part of the substrate-carbon into new biomass, in which carbon is more reduced than in CO_2_ (Rittmann and McCarty 2001). This reduces the amount of oxygen needed to degrade one unit of toluene under growth conditions. The *f*_*ox*_-values obtained for the different experiments fell in the range of 2.6–5.5 $\upmu {\text{mol}}_{\text{Tol}} \; \upmu {\text{mol}}_{{{\text{O}}_{ 2} }}^{ - 1}$, indicating carbon assimilation efficiencies of approximately 0.4–0.7. As can be seen from Fig. 3d, there is a positive relationship between *f*_*ox*_ and flow velocity. The faster the toluene is transported through the sediment, the less efficient is the substrate carbon converted into biomass. However, in total more toluene is degraded at higher flow rates and a higher standing stock of bacterial biomass is obtained (Fig. 3e). No pronounced differences were obtained for *f*_*ox*_ with changing toluene concentrations in the inflow (Fig. 3d).

In our second approach, the yield was calculated via the measured cell biomass produced from the toluene degraded. Based on microscopic measurements of cell dimensions (length and width), subsequent conversion into biovolume, and the assumption that carbon makes up 50% of the cell’s dry mass, the cell yields were converted into µmol_cell-carbon_ µmol_Tol_^−1^. Doing so, and taking into account attached cells, as well as cells suspended in pore-water and continuously flushed out of the sediment columns, we again estimated a range of carbon assimilation efficiencies of 0.4–0.7.

The carbon assimilation efficiencies in the batch experiments were found to be around 0.4–0.5 and could only be calculated via cell counts since there was a continuous supply of oxygen from the headspace in the batch bottles. Carbon assimilation efficiencies were therefore slightly lower in batch (0.4–0.5) than in the flow-through sediment microcosms (0.4–0.7). This is surprising, since high carbon assimilation efficiencies of > 0.5 are generally reported for batch and chemostat systems (Ho and Payne 1979; Payne and Wiebe 1978, and references therein).

Both yield estimates obtained in our experiments are subject to uncertainty. Estimation of cell carbon via cell measurements in the microscope and translation into cell carbon is based on a number of assumptions, such as the carbon content of the dry biomass. Conversely, to obtain *f*_*ox*_ values, we used mean oxygen values within the sediment columns ignoring the potential concentration gradients along the three points of measurement (bottom, middle, top; Fig. 1). The following additional points may affect the reliability and comparability of the yield estimates. First, the yield was calculated over the entire duration of the experiments, up to 3 days in the batch experiments and up to 9 days in the column experiments, including the stationary phase in the batch systems and the quasi steady-state phase in the microcosms. Second, in the batch experiments, the cultures received a single donation of substrate at *t*_0_ which then was continuously depleted, while in the flow-through systems substrate was supplied continuously causing a concentration gradient from the column inlet to the outlet. Third, in the batch system, cells generally stop growing when the substrate was depleted. In the flow-through systems, growth decelerated when the maximum cell density was reached, which is termed the carrying capacity in ecology (del Monte-Luna et al. 2004). Finally, if we consider that the substrate is first converted to intermediates which are subsequently utilized for assimilation and mineralization, the overall yield changes during the experiment and can only be deduced by fitting a model that account for a generic intermediate, unless all possible metabolites are monitored. Nevertheless, our experiments provide strong evidence that in flow-through systems cells attached to a solid matrix are more efficiently converting substrate carbon into biomass than suspended ones.

#### Suspended and attached cells and carrying capacity

Batch systems are closed and optimized systems containing only suspended bacteria, and thus don’t resemble the conditions in aquifers. The column systems are a step closer to field conditions as they represent flow-through systems containing both a mobile pore-water phase and an immobile sediment matrix with a solid-to-water ratio close to aquifer conditions (Hofmann et al. 2016). The distribution of cells between these two phases have been reported to depend on several factors including nutrient concentration in the pore-water, flow velocity, surface structure, and nutrient content of the sediment particles (Banfield and Hamers 1997; Banfield et al. 1999; Bennet et al. 2000, 2001; Carson et al. 2009; Ehrlich 1996; Marshall 1988; Mauck and Roberts 2007; Rogers and Bennet 2004; Tuschewitzki et al. 1992; Grösbacher et al. 2016). As a consequence, bacterial growth in flow-through sediment systems can only be monitored if changes in cell numbers are followed in both the attached and suspended populations. Cells that are continuously washed out may be a major contribution to the overall biomass balance. In our column systems, about 98–99% of the cells were found attached to the sediment at any time during the experiment; such numbers are also found in most aquifers (Alfreider et al. 1997; Griebler et al. 2002; Lehman et al. 2001a, b).

In all column experiments performed in this study, the amount of attached cells plateaued at a constant level after an initial growth phase, which indicates that the columns had a maximum carrying capacity for attached cells (Zhou et al. 2012). The carrying capacity showed an increasing trend with the amount of toluene degraded in the individual experiments. This suggests that the utilizable mass flux of substrate contributes to the control of the carrying capacity for attached bacteria. While the number of attached cells plateaued at concentrations of 0.9 × 10^8^ to 27 × 10^8^ cells mL_sed_^−1^ in the different experiments, 2 × 10^6^ to 4 × 10^6^ cells mL^−1^ were detected in the column outlets under stable experimental conditions. In fact, the ratio of attached to suspended cells was highest at the lowest substrate concentrations and vice versa, a pattern that is well known from aquatic sediments including aquifers (Harvey et al. 1984; Bengtsson 1989; Griebler et al. 2001, 2002).

Once the cells had reached the carrying capacity on the sediment, newly formed cells were released into the pore-water where they are transported and occasionally washed out of the columns. Over the course of the entire experiments (max duration of 9 days), this outwash corresponded to about 20–93% of all newly grown cells. An important finding was that growth-facilitated release of attached cells into the mobile phase was very strain-specific. While *P. putida* F1 cells growing on the sediment substantially released cells into the pore-water, fewer cells were released by the natural community and the fewest by the denitrifying strain *A. aromaticum* EbN1. A significant contribution from budding and detachment cells to the free floating cells in pore-water under growth conditions has been repeatedly observed in earlier studies (e.g., Clement et al. 1997; Murphy et al. 1997; Yolcubal et al. 2002; Jordan et al. 2004) and referred to as cell-division mediated transport by Murphy and Ginn (2000). The finding that the vast majority of cells is attached to the sediment surface suggests that suspended cells have only a minor impact on the overall contaminant degradation, which was confirmed by reactive-transport simulations. In our experiments, the residence time of the pore-water in the columns was rather short (between 6.5 and 38.5 min depending on the flow rate). Still, even if suspended cells do not significantly contribute to contaminant degradation at anywhere within an aquifer, the continuous release of new-grown cells from the sediment to the mobile aqueous phase, which has been observed in our study, increases the ability of bacteria to spread and colonize new sediment surfaces. This may be an important mechanism to establish a high biodegradation potential throughout an aquifer, which would be needed if hydrological fluctuations change the spatial distribution of the contaminants.

Microbes always grow towards the substrate source, even against strong currents. To reduce the effects of steep gradients on the quantification of turnover rates, we have chosen very small and short sediment columns. However, while we observed no significant gradient in cell numbers in most experiments, a distinct gradient in cell density was obvious in experiment B, with the highest cell density in the bottom part of the column near the inlet port for toluene and oxygen. The fact that the oxygen concentrations hardly differed between the bottom, middle, and top parts of the columns in all experiments indicates that microbial activity was indeed mainly restricted to the bottom (inflow) part of the columns, once a stable community of attached bacteria had developed. Interestingly, the continuous release of new cells mainly produced at the column inlet into the pore-water and its subsequent attachment to sediment particles downgradient, revealed a similar density of attached cells throughout the columns in most of the experiments. We may also not exclude the existence of steep small-scale biomass gradients at the column inlet that were beyond our spatial sampling resolution.

#### Reactive-transport modeling

The combined measurement of attached and suspended cells enabled us to develop a quantitative model, which explicitly accounts for the release of new-grown cells from the sediments to the mobile aqueous phase by the dynamic growth-dependent detachment rate *r*_*daughter*_. The reactive-transport simulations revealed that suspended cells were irrelevant for toluene degradation in the column experiments. Although it is well known that bacteria transported in porous media are important as seed banks in aquifers (Griebler et al. 2014) and play an important role in partitioning between the mobile aqueous phase and the sediment surface (e.g. Ginn et al. 2002; Tufenkji 2007; Scheibe et al. 2011; Zhou et al. 2012), the finding that the majority of bacteria in aquifers is attached to the sediment matrix led to the situation that the biomass catalyzing the breakdown of organic contaminants is usually treated as immobile species in reactive-transport models, and the presence of bacteria suspended in the mobile aqueous phase is neglected (e.g., Barry et al. 2002; Schirmer et al. 2000; Prommer et al. 2006, 2009). Moreover, the majority of studies on the transport of microorganisms in porous media was conducted under non-growth conditions and aimed at improving the understanding of the physical processes (e.g. straining and filtration) that govern microbial transport in porous media, which are important for the fate and behavior of pathogens in groundwater. A few studies, investigating the effect of biological processes on microbial transport in porous media, indicated that microbial growth strongly affects the partitioning of bacteria between the aqueous phase and the sediment surface in addition to physical processes (e.g., Clement et al. 1997; Murphy et al. 1997; Yolcubal et al. 2002; Jordan et al. 2004; Eckert et al. 2015). In all of these studies an increase in the number of suspended bacteria was observed after the addition of a growth substrate to the system.

While the results obtained in this study clearly highlight the flow velocity, the substrate concentration, and the electron-acceptor limitation as different drivers of microbial contaminant degradation in liquid batch and flow-through sediment systems, the data obtained from our lab experiments with single degrader strains should be translated to the field situation with caution. In situ, additional factors like food web interactions, grazing, or competition for resources as well as multiple limitations play an important role within natural microbial communities (Konopka 2000; Griebler and Lueders 2009; Griebler et al. 2014; Meckenstock et al. 2015). Numbers of attached bacteria as high as 10^8^ to 10^9^ cells mL^−1^ sediment as established in our column experiments are hardly observed in aquifers. Even at comparable high or even higher toluene concentrations, cell densities are typically 1–2 orders of magnitude lower (Winderl et al. 2008; Anneser et al. 2010). In our studies, the tested microbial community, obtained from natural aquifer sediments, degraded toluene at a slower pace than the specialized toluene-degrading strains *P. putida* F1 and *A. aromaticum* EbN1. This is most probably due to the fact that in natural communities, only a small fraction of the community is metabolically active while the majority is in an inactive resting state (Shade et al. 2012). Moreover, only a portion of the active cells in a community might be capable of utilizing petroleum hydrocarbons like toluene, which are toxic at elevated concentrations to many bacterial species (Castillo and Ramos 2007; Herzyk et al. 2013, 2017; Okpokwasili and Nweke 2005; Vecht et al. 1988). Assuming that the toluene-degrading species have a low overall abundance at the beginning of the experiment, the onset of pronounced toluene degradation is delayed until these toluene degrading species reach sufficient cell numbers. We should never expect that degraders under in situ conditions reach the carbon assimilation efficiencies and maximum growth rates observed in the lab. Nonetheless, our results are an important step towards a better understanding of ecological drivers of organic-contaminant biodegradation.

## Conclusions

A recent review by Meckenstock et al. (2015) highlights that many common concepts regarding degradation of organic contaminants by microbes in aquifers need considerable revision. One important aspect is that our lab-based knowledge, which is mainly derived from batch experiments, is of limited use in understanding and predicting processes in a natural, heterogeneous, complex, open flow-through sediment system. Our study underlines that degradation and associated growth rates are insufficiently predicted using laboratory batch experiments, which can lead to overestimating anticipated in situ biodegradation. Also, only flow-through sediment systems allow for an independent assessment of attached and suspended cells. As was found, attached bacteria are responsible for the majority of the observed biodegradation. While attached cells were mainly responsible for toluene degradation, the release of cells into the pore water causes permanent inoculation of the aquifer downstream. In consequence, the ratio of sediment to water is crucial when setting up laboratory experiments representative of field conditions. Finally, mass transfer limitation is an important process controlling toluene biodegradation that cannot be reproduced with laboratory batch experiments. We are convinced that mathematical models that simulate biodegradation and bacterial growth in aquifers will greatly be improved in their accuracy when they are calibrated by data derived from flow-through sediment microcosms and/or directly from field studies at appropriate spatial and temporal resolution.

## Electronic supplementary material

Below is the link to the electronic supplementary material.

Supplementary material 1 (DOCX 350 kb)
