# Supplementary material for: Contaminant concentration versus flow velocity: drivers of biodegradation and microbial growth in groundwater model systems
Source: Biodegradation. 2018 Feb 28;29(3):211–32. doi: 10.1007/s10532-018-9824-2 (PMC5943387; doi:10.1007/s10532-018-9824-2)
Supplement: Supplementary file 1 — Supplementary material 1 (DOCX 350 kb) [file 10532_2018_9824_MOESM1_ESM.doc]

**Contaminant Concentration versus Flow Velocity – Drivers of Biodegradation and Microbial Growth in Groundwater Model Systems**

Supplementary Information

**Michael Grösbacher**^a^**, Dominik Eckert**^b,c^**, Olaf A. Cirpka**^b^**, Christian Griebler***^a^

^a^Helmholtz Zentrum München - German Research Center for Environmental Health,

Institute of Groundwater Ecology, Ingolstädter Landstrasse 1, 85764 Neuherberg, Germany

^b^University of Tübingen, Center for Applied Geoscience, Hölderlinstrasse 12, 72074 Tübingen, Germany

^c^now at: Ingenieurgesellschaft Prof. Kobus und Partner GmbH, Heßbrühlstrasse 21D, 70565 Stuttgart, Germany

*Corresponding author: Dr. Christian Griebler, E-mail: griebler@helmholtz-muenchen.de

Phone: +49 (089) 31 87 25 64; Fax: +49 (089) 31 87 33 61

Helmholtz Zentrum München - German Research Center for Environmental Health,

Institute of Groundwater Ecology, Ingolstaedter Landstrasse 1, 85764 Neuherberg, Germany


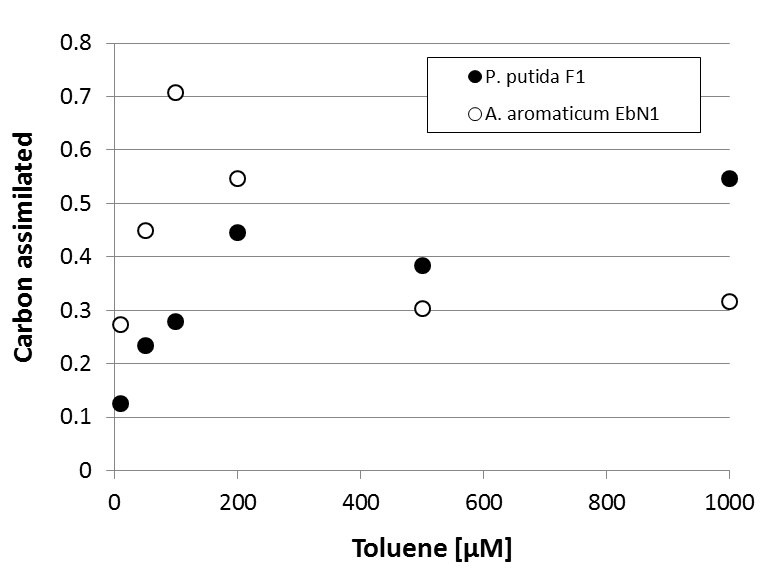


**Figure S1:** Substrate carbon converted into biomass carbon in batch experiments with the aerobic strain *P. putida* F1 and the nitrate reducing strain *A. aromaticum* EbN1 at varying initial concentrations of toluene. Toluene was the sole carbon and energy source.

**Figure S2:** Measured and simulated toluene concentrations and bacterial growth of *A. aromaticum* EbN1 over the course of batch experiments with different substrate starting concentrations.

**Figure S3:** Aerobic toluene degradation and growth of *P. putida* F1 in mini-sediment columns flow-through experiments at a continuous source concentration of 30 µM and a flow velocity of 1.8 m d^-1^ (Exp. D). Values are means of triplicate measurements ± SD.

**Figure S4:** Aerobic toluene degradation and growth of *P. putida* F1 in mini-sediment columns flow-through experiments at a continuous source concentration of 70 µM and a flow velocity of 0.6 m d^-1^ (Exp. A). Values are means of triplicate measurements ± SD.

**Figure S5:** Aerobic toluene degradation and growth of *P. putida* F1 at a continuous source concentration of 70 µM and a flow velocity of 1.8 m d^-1^ (Exp. B). Values are means of triplicate measurements ± SD.

**Figure S6:** Aerobic toluene degradation and growth of *P. putida* F1 at a continuous source concentration of 70 µM and a flow velocity of 3.6 m d^-1^ (Exp. C).Values are means of triplicate measurements ± SD.

**Figure S7:** Toluene degradation and growth of *A. aromaticum* EbN1 under nitrate reducing conditions at a continuous source concentration of 70 µM and a flow velocity of 1.8 m d^-1^. Values are means of triplicate measurements ± SD.

**Figure S8:** Carbon assimilation efficiency calculated from f_ox_ during different stages of the aerobic flow-through sediment column experiments with P. putida F1 and the natural aquifer microbial community.

**Figure S9:** Aerobic toluene degradation and growth of a natural aquifer microbial community at a continuous inflow concentration of 70 µM and a flow velocity of 1.8 m d^-1^. Values are means of triplicate measurements ± one standard deviation.

**Figure S10:** Comparison between the early stage growth patterns of attached and suspended bacterial populations during the different sediment column experiments.

**Appendix**

**A. Bacterial strains and growth medium**

*Pseudomonas putida* strain F1

The aerobic bacterium used was Pseudomonas putida strain F1, belonging to the class of the γ-Proteobacteria. It is a gram-negative, aerobic, motile bacterium (Chapelle, 2000; Madigan et al., 2003) with an average cell size of 1.5*1 µm. The optimum growth conditions were found to be at 25-30°C at a pH of 7.2-7.4.

*Aromatoleum aromaticum* strain EbN1

The anaerobic bacterium used was the denitrifyer Aromatoleum aromaticum strain EbN1, a gram-negative, facultatively anaerobic γ-Proteobacterium (Trautwein et al., 2008; Zink and Rabus, 2010) whose cells are rod-shaped, motile and have a size of about 0.6-0.8*1.5-2.5 µm. The optimum growth conditions are at 26-31°C and at a pH of 7.1-7.4 (Rabus and Widdel, 1994).

The growth medium

Both strains use almost the same medium (Widdel-Sweet-Medium) for growth, the only difference being that *Aromatoleum aromaticum* strain EbN1 needs anoxic conditions and a source of nitrate (50 mL of 1M NaNO_3_ L^-1^ medium). 1L of medium contained the following: 1g NaCl, 0.4g MgCl_2_*6H_2_O, 0.2g KH_2_PO_4_, 0.25g NH_4_Cl, 0.5g KCl, 0.15g CaCl_2_*2H_2_O, 0.2% trace element solution SL-10 (Widdel et al., 1983), 0.2% selenite-tungsten solution (Tschech and Pfennig, 1984), 0.1% 7 vitamin solution (Widdel and Bak, 1992). The medium was autoclaved at 121°C for 30 min, and gas exchange was performed using N_2_/CO_2_ gas in an 80:20 ratio. Once the medium has cooled to room temperature, 30 mL of 1M NaHCO_3_ buffer and 0.5 mL of 1M Na_2_SO_4_ were added. Afterwards, the pH was adjusted to around 7.2 to 7.4. The medium was then filled into several 120 mL serum bottles, approximately 50-70 mL per bottle. The bottles were closed with viton stoppers, and the medium was stored in the dark at room temperature. When preparing an anaerobic medium, the bottles were exposed to N_2_/CO_2_ gas for a couple minutes after filling them with medium, to remove any remaining oxygen.

**B. Experiment with a natural community and the alternative substrate α-Ketoglutarate**

Another column experiment with the same natural community was performed using α-Ketoglutarate as a substrate to compare the growth and degradation potential of the same natural community between two different carbon sources. The concentration of α-Ketoglutarate was constantly measured using the α-Ketoglutarate Colorimetric/Fluorimetric Assay Kit from BioVision. The natural community degraded α-Ketoglutarate much faster than toluene. Two days after the start of the experiment, no α-Ketoglutarate was measurable in the outflow anymore (data not shown). Contrary to the experiment with toluene as the sole carbon source, degradation started instantly along with immediate and fast bacterial growth, most likely because α-Ketoglutarate is not toxic and a highly sought-after substrate for many bacterial species.
